# Supplementary material for: Alpine endemic spiders shed light on the origin and evolution of subterranean species
Source: PeerJ. 2015 Nov 3;3:e1384. doi: 10.7717/peerj.1384 (PMC4699788; doi:10.7717/peerj.1384)
Supplement: Supplemental Information 1 [file peerj-03-1384-s001.pdf]

SUPPLEMENTARY MATERIALS

Alpine endemic spiders shed light on the origin and evolution of subterranean species

Stefano Mammola [1], Marco Isaia [1] & Miquel A. Arnedo [2]

1. Department of Life Sciences and Systems Biology, University of Turin, Via Accademia Albertina, 13, 10100, Torino, Italy; stefanomammola@hotmail.it; marco.isaia@unito.it

2. Biodiversity Research Institute and Departament de Biologia Animal, Universitat de Barcelona, Av. Diagonal 643, 08028, Barcelona, Spain; marnedo@gmail.com

Ecological Niche modelling

We relied on ecological niche modeling (ENM) tools to model the ancestral distribution of the target species. ENMs have been extensively use to identify Pleistocene refugia (e.g. Waltari et al., 2007; Rodriguez-Sanchez & Arroyo, 2008; Planas *et al.*, 2014), since they facilitate the correlation of occurrence data with presumed environmental predictors, and the projection of such relationships to different time-periods and/or geographic spaces (Elith *et al.*, 2006).

Occurence points and sampling-bayas grid

An exhaustive bibliographic investigation was conducted in the scientific literature (Brignoli, 1971, 1972, 1985; Thaler, 1976; Hormiga, 1994; Arnò & Lana, 2005; Isaia *et al.*, 2011 among others) to recollected the occurrence records for *Pimoa rupicola* (Simon, 1884) (Araneae, Pimoidae). Localities for which we were not able to obtain precise latitude/longitude coordinates were excluded from the ENM analysis. Material of *P. rupicola* cited in several works actually belong to the potential new species (*Pimoa* "n.sp.") identified in this study. Localities of *P.* n.sp. were reassigned on the base of the reexamination of the original material cited in literature (when adult spiders were available), on the genetic data and on geographic base. The map of occurrence point for which we were able to obtain precise latitude/longitude is reported in Fig. 1.

In a next step, we designated a sampling bias grid (Phillips *et al.*, 2009; Syfert *et al.*, 2013) to correct our occurrence dataset for potential spatial autocorrelation and haphazard sampling (i.e. variation in sampling effort). Each cell of the grid was constructed with a width of 30 x 30 arc-seconds, corresponding to the resolution of the present climate rasters. In each cell of the grid we deleted all the occurrence points of *Pimoa* but one. Doing so, we cleaned our dataset from

duplicates (see Newbold, 2010) and hence the over-expression of certain environmental variables (i.e. given the resolution of the raster, spatially clumped localities are characterized by equal climatic parameters). At the same time, the cleaning of the dataset from clustered points allowed us to compute the model with a more geographically scattered set of occurrence points across the landscape (see Yackulic *et al.*, 2013).

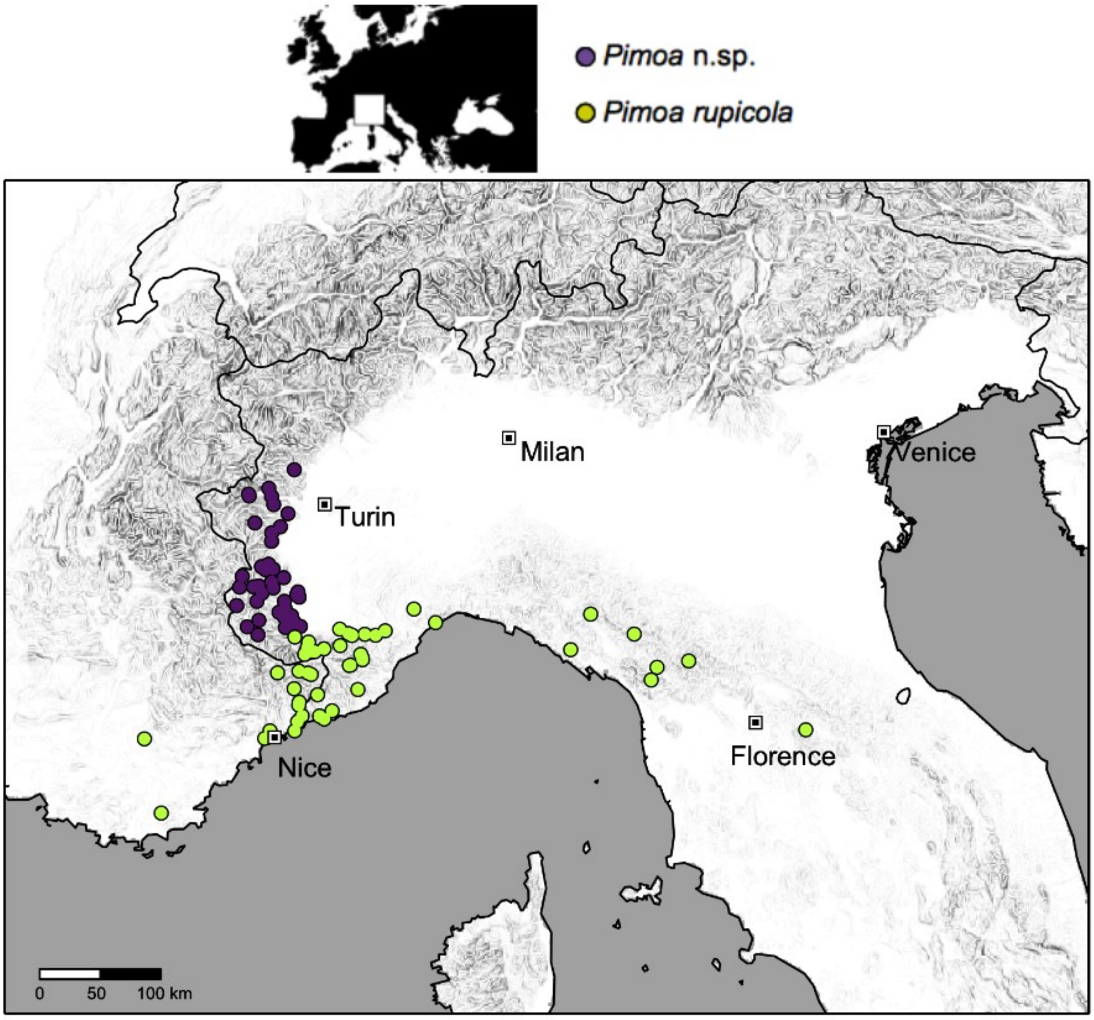

Fig. 1- Occurrence points for *Pimoa rupicola* and *P.* n.sp.

Environmental variables

We obtained present-day climatic data (19 "Bioclim variables", tab. 1) and altitude a.s.l. from the WorldClim website (www.worldclim.org), at a spatial resolution of 30 arc-seconds (~1 km). We chose this resolution because of the sub-continental distribution of the two *Pimoa* lineages. The twenty environmental variables were stacked in a single raster via the command *stack*

implemented in the *Raster* R package (Hijmans, 2014).

We obtained downscaled and calibrated (bias corrected) Paleoclimatic data for the Last Glacial Maximum (~22,000 years ago; hereinafter LGM) from three different simulations available from Global Climate Models (GCMs): Community Climate System Model (CCSM), MIROC-ESM and the New Earth system model of the Max Planck Institute for Meteorology (MPI-ESM-P). Reconstruction were made available by the CMIP5 (Coupled Model Intercomparison Project phase 5; online at: http://cmip-pcmdi.llnl.gov/cmip5), at a resolution of 2.5 minutes. Although the LGM climate is relatively well known (Ivy-Ochs *et al.*, 2008), we used simulations from different sources to account for unavoidable uncertainty associated to paleo-reconstructions (Kageyama et al., 2001). Similarly, we did not downscale the LGM rasters to obtain the same spatial resolution of the present-day climatic rasters (i.e. 30 arc-sec). These predictions, in any case, should be considered as broad estimates of potential past conditions given the uncertainties associated (e.g. Planas *et al.*, 2014).

Table 1 - Climate variables from the WorldClim website (www.worldclim.org)

Variable	Description	Unit
Bio1	Annual Mean Temperature	°C
Bio2	Mean Diurnal Range (Mean of monthly (max temp - min temp))	°C
Bio3	Isothermality (BIO2/BIO7) (* 100)	°C
Bio4	Temperature Seasonality (standard deviation *100)	°C
Bio5	Max Temperature of Warmest Month	°C
Bio6	Min Temperature of Coldest Month	°C
Bio7	Temperature Annual Range (BIO5-BIO6)	°C
Bio8	Mean Temperature of Wettest Quarter	°C
Bio9	Mean Temperature of Driest Quarter	°C
Bio10	Mean Temperature of Warmest Quarter	°C
Bio11	Mean Temperature of Coldest Quarter	°C
Bio12	Annual Precipitation	mm
Bio13	Precipitation of Wettest Month	mm
Bio14	Precipitation of Driest Month	mm
Bio15	Precipitation Seasonality (Coefficient of Variation)	mm
Bio16	Precipitation of Wettest Quarter	mm
Bio17	Precipitation of Driest Quarter	mm
Bio18	Precipitation of Warmest Quarter	mm
Bio19	Precipitation of Coldest Quarter	mm
Alt	Altitude a.s.l	m

Collinearity

For each of occurrence point, we extracted the punctual values of the 20 explanatory variables from the stacked present-day climatic raster (Fig. 2). Pairwise Pearson correlations (r) among the different extracted covariates evidenced a high level of inter-correlation between most of the Bioclimatic variables extracted for each of the occurrence points. Collinearity was handled by dropping, one by one, the Bioclimatic covariates, until a set of un-collinear covariates was obtained. We used the variance inflation factors values (VIFs; Zuur et al., 2009, 2010) to select the covariates. The final set of explanatory variables introduced in the ENM model consisted of 3 variables, namely Annual mean temperature (Bio1), Temperature annual range (Bio7) and Mean temperature of the driest quarter (Bio 9).

Figure 2 - Boxplots showing the range of climatic parameters extracted from the 3 Bioclim variables introduced in the ENMs.

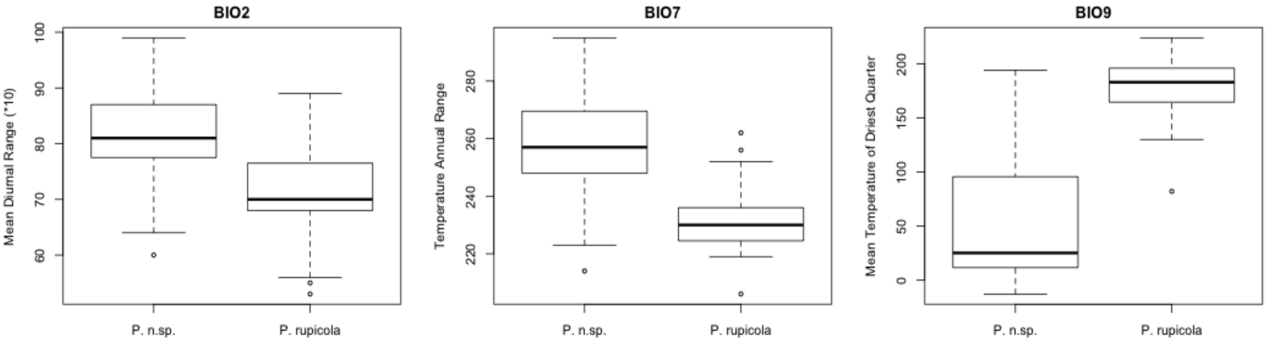

Algorithm and model calibration (M region)

The MaxEnt algorithm (Phillips *et al.*, 2006) was chosen because it does not require the use of absence points, which avoids the problems associated to unreliable absence record (e.g. Jiménez-Valverde *et al.*, 2008). Additionally, comparative studies have been shown that MaxEnt outperforms other ENM/SDM techniques (see Elith *et al.*, 2006). Firstly, we computed the model on the set of non-collinear variables selected after data exploration (present climate) and on the occurrence points. We computed two separated ENMs, one for *Pimoa* n.sp. and one for *P. rupicola*, respectively. ENMs were calibrated within the M region (Barve *et al.*, 2011), i.e. a geographic area that we hypothesized has been accessible to the two species over their

evolutionary history (see Saupe et al., 2012 for a detailed discussion on the topic). The M region was calculated a priori, by buffering the occurrence records of *Pimoa* n.sp. and *P. rupicola* by 70 km, the estimated area that is covered by the dispersal capability of the species.

Partition Finder results

Partition Finder (Lanfear *et al.*, 2012) selected the fullcodon as the best partition scheme for the alignments of both species. The best models for each partition are reported in the Tab. 2.

Table 2 - Best model selected for each subset partition for *Pimoa* and *Troglohyphanes* alignments according to lowest AIC in Partition Finder (Lanfear *et al.*, 2012).

Pimoa n. sp. - *P. rupicola*

Gene	Partitions	Best Model
cox1	1	K81uf+G
cox1	2	HKY+I
cox1	3	HKY+I
ITS-2	4	JC+I
ITS-2	5	K80+I
ITS-2	6	JC
ITS-2	7	TVMef+G
ITS-2	8	K80+I
ITS-2	9	JC+I
ITS-2	10	K81+I

Troglohyphantes

Gene	Partitions	Best Model
cox1	1	TrN
cox1	2	TrN+I
cox1	3	F81
ITS-2	4	JC
ITS-2	5	JC
ITS-2	6	JC
ITS-2	7	JC
ITS-2	8	JC
ITS-2	9	K80

Literature cited

Arnò, C. & Lana, E. (2005) Ragni cavernicoli del Piemonte e della Valle d'Aosta. Associazione Gruppi Speleologici Piemontesi, Regione Piemonte, Ed. "La Grafica Nuova", Torino, 265 p.

Barve, N., Barve, V., Jiménez-Valverde, A., Lira-Noriega, A., Maher, S.P., Peterson, A.T., Soberón, J. & Villalobos, F. (2011) The crucial role of the accessible area in ecological niche modeling and species distribution modeling. Ecological Modeling, 222, 1810–1819.

Brignoli, P.M. (1971) Note su ragni cavernicoli italiani (Araneae). Fragmenta entomologica, 7 (3), 129-229.

Brignoli, P.M. (1972) Catalogo dei ragni cavernicoli italiani. Quaderni di Speleologia del Circolo Speleologico Romano, 20, 1-211.

Brignoli, P.M. (1985) Aggiunte e correzioni al «Catalogo dei Ragni cavernicoli italiani». Mem. Mus. civ. St. nat. Verona, II (4), 51-64.

Elith, J., Graham, H., Anderson, P., Dudík, M., Ferrier, S., Guisan, A., Hijmans, J., Huettmann, F., Leathwick, R., Lehmann, A., Li, J., Lohmann, G., Loiselle, A., Manion, G., Moritz, C., Nakamura, M., Nakazawa, Y., Overton, C.M., Townsend Peterson, A., Phillips, J., Richardson, K., Scachetti- Pereira, R., Schapire, E., Soberón, J., Williams, S., Wisz, S. & Zimmermann, E. (2006) Novel methods improve prediction of species' distributions from occurrence data. Ecography, 29, 129-151.

Ivy-Ochs, S., Kerschner, H., Reuther, A., Preusser, F., Heine, K., Maisch, M., Kubik, P. W. and Schlüchter, C. (2008) Chronology of the last glacial cycle in the European Alps. Journal of Quaternary Science, 23, 559-573.

Jiménez-Valverde, A., Lobo, J.M. & Hortal, J. (2008) Not as good as they seem: the importance of concepts in species distribution modelling. Diversity and Distributions, 14, 885-890.

Kageyama, M., Peyron, O., Pinot, S., Tarasov, P., Guiot, J., Joussaume, S. & Ramstein, G. (2001) The Last Glacial Maximum climate over Europe and western Siberia: a PMIP comparison between models and data. Climate Dynamics, 17, 23-43.

Newbold, T. (2010) Applications and limitations of museum data for conservation and ecology, with particular attention to species distribution models. Progress in Physical Geography, 34, 3-22.

Oliveira G., Rangel T. F., Lima-Ribeiro M.S., Terribile L.C. & Diniz-Filho, J.A.F. (2014) Evaluating, partitioning, and mapping the spatial autocorrelation component in ecological niche modeling: a new approach based on environmentally equidistant records. Ecography, 37, 637-647.

Phillips, S.J., Anderson, R.P. & Schapire, R.E. (2006) Maximum entropy modeling of species geographic distributions. Ecological Modelling, 190, 231-259.

Phillips, S.J., Dudik, M., Elith, J., Graham, C.H., Lehmann, A., Leathwick L. & Ferrier S. (2009) Sample selection bias and presence-only distri- bution models: implications for background and pseudo-absence data. Ecological Applications 19, 181-197.

Planas E., Saupe E.E., Lima-Ribeiro M.S., Peterson A.T., Ribera C., 2014. Ecological niche and phylogeography elucidate complex biogeographic patterns in Loxosceles rufescens (Araneae, Sicariidae) in the Mediterranean Basin. BMC evolutionary biology, 14:195.

Rodriguez-Sanchez, F. & Arroyo J. (2008) Reconstructing the demise of Tethyan plants: climate-driven range dynamics of Laurus since the Pliocene. Global ecology & Biogeography, 1-11.

Saupe E.E., Barve, V., Myers, C.E., Soberón, J., Barve, N., Hensz, C.M., Peterson, A.T., Owens, H.L. , Lira-Noriega, A. (2012) Variation in niche and distribution model performance: The need for a priori assessment of key causal factors. Ecological Modelling, 11-12, 237-238.

Syfert, M.M., Smith, M.J. & Coomes, D.A. (2013) The Effects of Sampling Bias and Model Complexity on the Predictive Performance of MaxEnt Species Distribution Models. PLoS ONE, 8(2), e55158. doi:10.1371/journal.pone.0055158

Isaia, M., Paschetta, M., Lana, E., Pantini, P., Schőnhofer, A.L., Christian, E. & Badino, G. (2011) *Subterranean arachnids of the Western Italian Alps (Arachnida: Araneae, Opiliones, Palpigradi, Pseudoscorpiones).* Monografie XLVII, Museo Regionale di Scienze Naturali, Torino, 325 p.

Lanfear, R., Calcott, B., Ho, S.Y.W. & Guindon, S. (2012) *Partitionfinder: combined selection of partitioning schemes and substitution models for phylogenetic analyses*. Molecular Biology and Evolution, **29**, 1695-1701.

Thaler, K. (1976). Two remarkable relict arachnids from northern Italy: *Sabacon simoni* Dresco (Opiliones: Ischyropsalididae), *Louisfagea rupicola* (Simon) (Araneae: Tetragnathidae). Bulletin of the British Arachnological Society, **3,** 205-210.

Waltari, E., Hijmans, R.J., Peterson, A.T., Nyári, Á.S., Perkins, S.L. & Guralnick, R.P. (2007) Locating Pleistocene Refugia: Comparing Phylogeographic and Ecological Niche Model Predictions. PLoS ONE, 2(7), e563. doi:10.1371/journal.pone.0000563

Yackulic, C.B., Chandler, R., Zipkin, E.F., Royle, J.A., Nichols J.D., Campbell Grant, E.H. & Veran, S. (2013) Presence-only modelling using MAXENT: when can we trust the inferences? Methods in Ecology and Evolution, 4, 236–243.

Zuur, A.F., Ieno, E.N. & Elphick, S.C. (2010) A protocol for data exploration to avoid common statistical problem. Methods in Ecology and Evolution, 1, 3-14.

Zuur, A.F., Ieno, E.N., Walker, N.J., Savaliev, A.A. & Smith, G.M (2009) Mixed effect models and extensions in ecology with R. Ed. Springer, Berlin, 574 p.
